# The complete mitochondrial genome of the Amur soft-shelled turtle (*Pelodiscus maackii* Brandt, 1858), from South Korea

**DOI:** 10.1080/23802359.2022.2051759

**Published:** 2022-03-15

**Authors:** Hae-jun Baek, Philjae Kim, Young-Chae Kim, Areum Kim, Suhwan Kim, Mi-Sook Min, Hang Lee

**Affiliations:** a Conservation Genome Resources Bank for Korean Wildlife (CGRB) and Research Institute for Veterinary Science College of Veterinary Medicine, Seoul National University, Seoul, South Korea; b Invasive Alien Species Research Team, Bureau of Survey and Safety Research, National Institute of Ecology, SeoCheon, Chungcheongnam-do, South Korea; c Mammal Research Team, Research Center for Endangered Species, National Institute of Ecology, YeongYang, Gyeongsangbuk-do, South Korea; d Department of Life Sciences, College of Natural Science, Yeungnam University, Gyeongsan, Gyeongsangbuk-do, South Korea

**Keywords:** *Pelodiscus maackii*, Amur soft-shelled turtle, Korean peninsula, complete mitogenome

## Abstract

In this study, we use a specimen from wild-caught individual to determine the complete mitochondrial genome of the Amur soft-shelled turtle (*Pelodiscus maackii*). The complete mitogenome of *P. maackii* has 16,258 bp in length and consists of 13 protein-coding genes (PCGs), 22 tRNAs, two rRNAs, and one control region. The arrangement of genes of *P. maackii* is identical with previously reported mitogenomes in the family Trionychoidea. According to our result, the ML tree for the phylogenetic reconstruction revealed that the individuals used in present study is closely related with the previously reported sequences of *P. sinensis* (AY962573 and MG431983) in p-distance 0.7% and 2.5%.

The genus *Pelodiscus* is comprised of five species worldwide. In South Korea, two species of *Pelodiscus* are distributed, Amur soft-shelled turtle (*Pelodiscus maackii* Brandt, 1858) and Chinese soft-shelled turtle (*Pelodiscus sinensis* Wiegmann, 1835). *P. sinensis* and *P. maackii* are commonly confused in species-level identification because of their high morphological similarities. However, there are several morphological keys to distinguish (Chang et al. 2012; Farkas et al. 2019). Generally, *P. maackii* is known to have longer carapace length than *P. sinensis* and *P. maackii* has a no pattern and unmarked plastron with white to straw yellow. Besides, *P. sinensis* has a no pattern or relatively small, faint round to oval dark markings with snow white to pinkish white (Farkas et al. 2019). Even though *P. sinensis* is known as introduced species (Chang et al. 2012), the definite status of *Pelodiscus* species distributed in Korean peninsula is still controversial. So far, previous studies on *Pelodiscus* species in South Korea are very insignificant, especially in genetic studies, and the study on the mitogenome of *P. sinensis* using specimen collected from soft-shelled turtle farm is the only research conducted (Jung et al. 2006). Therefore, in this study, we conduct mitogenome research on the specimen collected in the natural state to clarify the species distribution state of Korean soft-shelled turtle and to confirm the phylogenetic status with related species.

One *P. maackii* (specimen voucher: TOR34) sample was collected from Ogye-ri (N35.009094° E126.865442°), Naju-si, Jeollanam-do, South Korea. A frozen corpse was taken and stored at −24 °C. Total genomic DNA was extracted using Qiagen DNeasy Blood and Tissue kit (Qiagen Korea Ltd, Seoul, South Korea), following the manufacturer’s instruction. The specimen and total genomic DNA are deposited at Invasive Alien Species Research Team of National Institute of Ecology (Hae-jun Baek, inshore72@nie.re.kr). The complete mitochondrial DNA sequence was analyzed on a Hiseq2000 platform using Next-Generation Sequencing (NGS) technique (Illumina, San Diego, CA). As a result, the complete mitogenome of *P. maackii* (NCBI) is comprised of a 16,258 bp nucleotide. The overall PCGs nucleotide compositions found to be 35% A, 27.6% T, 26.6% C, 10.6% G and 37.3% of GC content. Eleven out of the thirteen PCGs (ND1, ND2, COX2, ATP8, ATP6, COX3, ND3, ND4L, ND4, ND5, CytB) contained ‘ATG’ as start codon. The two remaining PCGs, COX1 and ND6, contained ‘GTG’ and ‘ATA’ as start codon respectively. There were five types of stop codon found as follows: T (COX3); TAG (ND1, ND2, ND3); TAA (COX2, ATP8, ATP6, ND4L, ND4, ND5, CytB); AGA (COX1); AGG (ND6).

**Figure 1.**
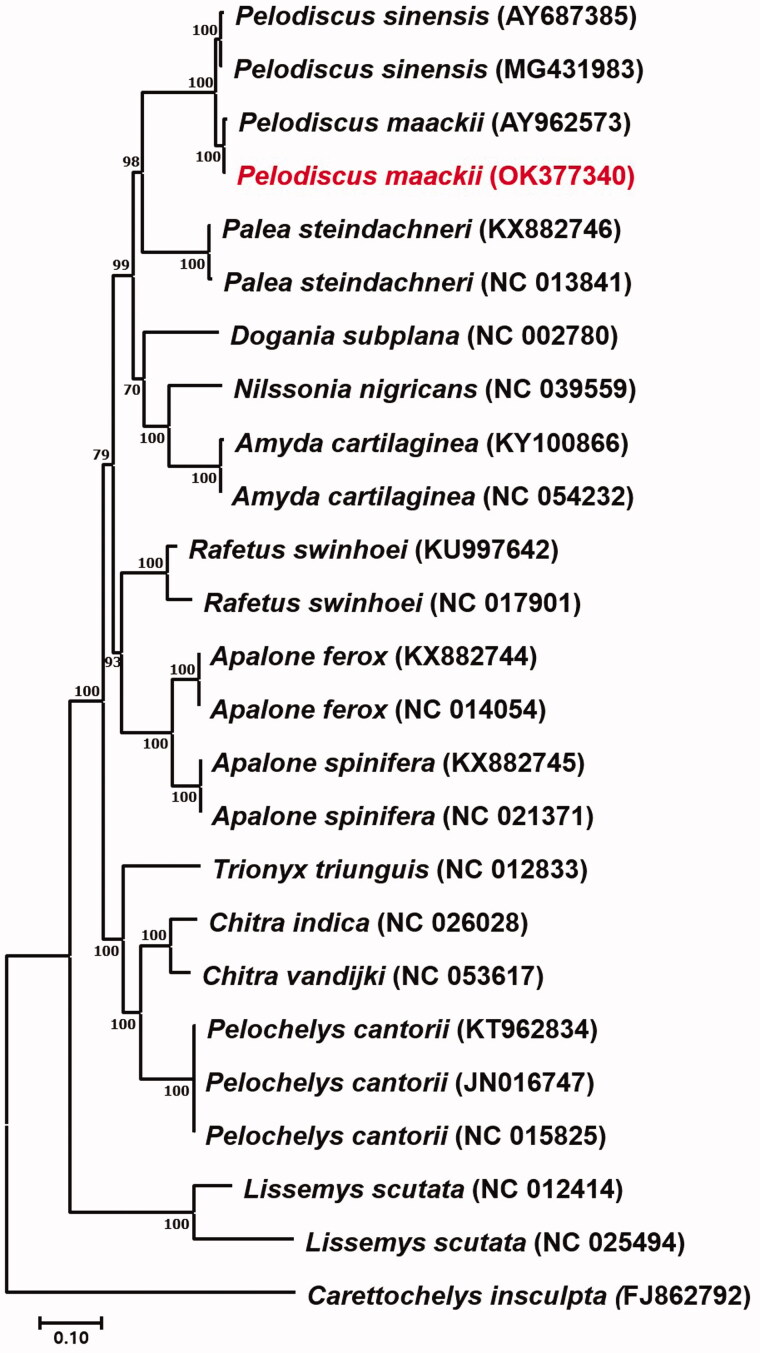
Maxium-likelihood (ML) tree based on analysis of 13 protein coding genes of 23 mitogenomes of Trionychoidea species, including *P. maackii* and outgroup (*Carettochelys insculpta* FJ862792), with GTR + I + G and bootstrapping (1000 replication).

To reconstruct the phylogenetic tree for *P. maackii*, 14 related species in the same superfamily Trionychoidea were used and the *Carettochelys insculpta* (FJ862792) belonging to Carettochelyidae was used as outgroup. DNA molecular phylogeny was reconstructed with 1,000 replication of bootstrapping by maximum likelihood (ML) methodologies with MEGA 7.0 (Kumar et al. 2016). The best fit model was chosen on the basis of Akaike information criterion (AIC) as formed in ModelTest-NG v0.1.6 in CIPRES (Miller et al. 2010), and GTR + I + G model was selected. For the phylogenetic reconstruction of 14 related species in Trionychoidea was well supported (BP >70%) and performed monophyly. Our findings from the present study coincide with the results from Li et al. (2017), which uses complete mitogenome of 14 species in Trionychoidea with minor difference caused by different methodologies used.

As a result, the specimen of *P. maackii* used in the present study is distinct, but almost identical with *P. sinensis* (AY962573) from the soft-shelled turtle farm in South Korea with a 0.7% of genetic distance (Figure 1). The other *P. sinensis* (MG431983) from China showed a 2.5% of genetic difference with the specimen used. The mean interspecific divergence in the genus *Pelodiscus* is 2–3% (Yang et al. 2011; Suzuki and Hikida 2014), which is relatively low compared to the other genera in Trionychoidea. Since the specimen of *P. sinensis* (AY962573) used in the present study has been re-separated into *P. maackii* by Fritz et al. (2010) using molecular makers and considering the finding of this research showing a similar result, it is reasonable to identify *P. sinensis* (AY962573) as *P. maackii*. This research is the first and only mitogenome study conducted on wild *P. maackii* in South Korea. Therefore, it is expected to make a great contribution to providing basic genetic data for the future studies of *P. maackii*.

## Ethical consideration and permissions

The experiment was conducted in accordance with the standard operating guidelines of the animal testing and/or laboratory animal related committee (IACUC) which is published by Animal and Plant Quarantine Agency and Ministry of Food and Drug Safety of South Korea. According to the IACUC ‘Even if vertebrates are available, experiments using only animal carcasses are not subject to the deliberation category of the Institutional Animal Care and Use Committee.’ In case dead cadavers were used for present study, Ethics approval is not applicable.

## Author contributions

Conception and design: Kim YC, Kim AR, Min MS, Lee H; analysis and interpretation of the data: Baek HJ, Kim PJ, Kim SH; the drafting of the paper: Baek HJ, Kim PH, Min MS; revising it critically for intellectual content; Kim PJ, Baek HJ, Lee H; the final approval of the version to be published: Baek HJ, Kim PJ. All authors agree to be accountable for all aspects of the work.

## Data Availability

The genome sequence data that support the findings of this study are openly available in GenBank of NCBI at (https://www.ncbi.nlm.nih.gov/) under the accession no. OK377340. The associated BioProject, SRA and Bio-Sample numbers are PRJNA768941, SRX12511000 and SAMN22071900 respectively.
